# A quantitative evaluation of multiple biokinetic models using an assembled water phantom: A feasibility study

**DOI:** 10.1371/journal.pone.0189244

**Published:** 2017-12-21

**Authors:** Da-Ming Yeh, Ching-Yuan Chen, Jing-Fa Tang, Lung-Kwang Pan

**Affiliations:** 1 Department of Diagnostic Radiology, Chung Shan Medical University Hospital, Taichung, Taiwan, ROC; 2 School of Medical Imaging and Radiological Sciences, Chung Shan Medical University, Taichung, Taiwan, ROC; 3 Graduate Institute of Radiological Science, Central Taiwan University of Science and Technology, Takun, Taichung, Taiwan, ROC; 4 Department of Nuclear Medicine, Buddhist Tzu Chi General Hospital, Taichung Branch, Taichung, Taiwan, ROC; Universidad Rey Juan Carlos, SPAIN

## Abstract

This study examined the feasibility of quantitatively evaluating multiple biokinetic models and established the validity of the different compartment models using an assembled water phantom. Most commercialized phantoms are made to survey the imaging system since this is essential to increase the diagnostic accuracy for quality assurance. In contrast, few customized phantoms are specifically made to represent multi-compartment biokinetic models. This is because the complicated calculations as defined to solve the biokinetic models and the time-consuming verifications of the obtained solutions are impeded greatly the progress over the past decade. Nevertheless, in this work, five biokinetic models were separately defined by five groups of simultaneous differential equations to obtain the time-dependent radioactive concentration changes inside the water phantom. The water phantom was assembled by seven acrylic boxes in four different sizes, and the boxes were linked to varying combinations of hoses to signify the multiple biokinetic models from the biomedical perspective. The boxes that were connected by hoses were then regarded as a closed water loop with only one infusion and drain. 129.1±24.2 MBq of Tc-99m labeled methylene diphosphonate (MDP) solution was thoroughly infused into the water boxes before gamma scanning; then the water was replaced with de-ionized water to simulate the biological removal rate among the boxes. The water was driven by an automatic infusion pump at 6.7 c.c./min, while the biological half-life of the four different-sized boxes (64, 144, 252, and 612 c.c.) was 4.8, 10.7, 18.8, and 45.5 min, respectively. The five models of derived time-dependent concentrations for the boxes were estimated either by a self-developed program run in MATLAB or by scanning via a gamma camera facility. Either agreement or disagreement between the practical scanning and the theoretical prediction in five models was thoroughly discussed. The derived biokinetic model represented the metabolic mechanism in the human body and helped to solidify the internal circulatory system into concert with numerical verification.

## Introduction

This work examines the feasibility of quantitatively evaluating multiple biokinetic models and establishes the validity of the different compartment models using an assembled water phantom. Most phantoms are customized either to survey the imaging system for increasing diagnostic accuracy or to survey the exposure dose for quality assurance. Phantoms are more convenient in these contexts compared to the human body because of the high reproducibility of measurements and easy manipulation in manual operations. However, few phantoms are made to imply the multi-compartment biokinetic models. This is because the cumbersome calculations required to solve the biokinetic models and the time-consuming verifications of the obtained solutions have significantly impeded the progress over the past decade [1]. A well-defined biokinetic model can assist the medical physicists in estimating internal dose or predicting the time-dependent quantity of radio-labeled complexes in crucial organs from the clinical viewpoint.

Most reported biokinetic models are limited in either theoretical simulation or computational calculation for their difficult verifications from the practical standpoint. Nevertheless, in real-world applications, most commercialized phantoms are either too simple or too unique to quantitatively verify the biokinetic models. Sometimes a phantom is customized as an elliptical box with only one infusion and a drain hose; the dynamic metabolic mechanism is suggested by changing the amount of feed-water input [2, 3, 4]. As an alternative to this method, Chiang [1] proposed a unique water phantom to simulate five different biokinetic models by adjusting the water paths among the various water chambers. The adjustable path setting can be customized to fulfill a specific demand, although the limited combination of multiple path settings still has further applications. In this work, seven water boxes, in four different sizes, are freely assembled to represent numerous biokinetic models for satisfying medical or hygienic needs. The modular box has six connectors along the rectangular side. Therefore, each box can be linked to a maximum of six boxes, on the as-needed basis. The silicon-based hose adopted herein has two functions. It can function as a regular hose, and it also has a one-way valve, so that water can only flow in one assigned direction. This unique feature of the hose helps verify the biokinetic model with a feedback path, such as in the thyroid I-131 model [5].

To further explore and solidify the metabolic mechanism inside the human body, Giussani defined a revised biokinetic model with six compartments to simulate the ^18^F-Choline in ten prostate cancer patients and used in vivo measurements for verification [6], while Chen verified the thyroid biokinetic model of administering I-131 to five thyroidectomy patients [5]. In another study, Hsu validated the gastro-intestine tract (GI tract) biokinetic model using 24 healthy volunteers [7]. After the Fukushima nuclear disaster, Tani adopted a well-defined biokinetic model for calculating the I-131 concentration in breast milk [8], while in a recent study Kenneth used a simplified model for predicting inorganic cobalt levels in whole blood and urine [9]. However, most of the reported data were barely verified because of the limited application from most commercial available phantoms.

In contrast, in this study, five models from many possible combinations were assembled and evaluated by seven water boxes to represent five biokinetic models. Two out of the five were organized with a feedback path, and each combination of multiple boxes was filled entirely with 129.1±24.2 MBq of Tc-99m labeled methylene diphosphonate (MDP) solution before being scanned. During gamma camera scanning, the radioactive solution inside the phantom was gradually replaced by the newly fed deionized water, and the time-dependent radioactivity changes were derived in each specific box. The acquired data can be interpreted as an integrated effect of both the radiological half-life and the biological half-life. The biological half-life was manipulated by changing the flow rate of the deionized feed-water. A program was developed in MATLAB to simulate the theoretical performance of time-dependent Tc-99m radioactivity changes according to different biokinetic models. The practical measurements and theoretical estimations are discussed and elaborated. A specific dimensionless index, the agreement (AT) or (AT_Wi_), is proposed to quantify the deviations among the comparison groups.

## Experiment

### Seven box water phantom

**Fig 1:**
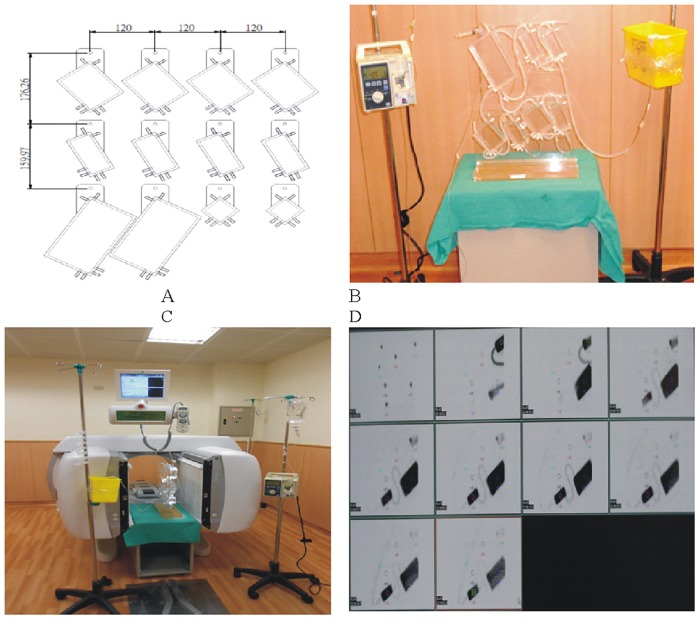
The water phantom as adopted in this work. (A) the designation chart of the boxes with four different sizes; 1/2, 1, 2, and 4 unit. (B) the well-assembled water phantom. The infusion hose was connected to a medical infusion pump (IV pump, Hospira plum XL series) and the drain hose connected to a container. It should be noted that the height of the drain hose needed to be maintained at the same height as the infusion port; this was essential to maintain the water circulation balance inside the phantom. (C) The assembled water phantom set between two collimators of the gamma camera during the data acquisition process. (D) Ten consecutive diagrams were acquired directly from the gamma camera scanning. The plot was created from one of the five models derived in this work. The first photo was background scanning before the initial time begun (on the left-side of the first row), the others were taken from 12 minutes of scanning for every 15 minutes within consecutive 2 hour periods.

Four different box sizes were made from 5 mm thick acrylic material (PMMA). The boxes were in the following sizes: 1/2 unit (active volume 4×4×6 = 64 c.c.), 1 unit (4×4×9 = 144 c.c.), 2 unit (4×7×9 = 252 c.c.), and 4 unit (4×7×21.9 = 612 c.c.). Each box has six sockets along the rectangular side to easily connect the silicon-based hose to other boxes. The adopted hose has two different functions. It can function regularly, and it also has a one-way valve that allows water to pass through from one side. This unique feature has helped to verify the biokinetic model with a feedback path from the medical physicist’s viewpoint. Seven boxes were assembled as a closed circular loop with only one infusion or drain port. One box was four units, one was 1/2 unit, two were two units, and three were 1 unit, and the total volume of the assembled water phantom was 1612 c.c. (612+64+2×252+3×144 = 1612 c.c.). Fig 1 depicts the water phantom as adopted in this work, (A) the designation chart of the boxes with four different sizes; 1/2, 1, 2, and 4 unit. (B) the well-assembled water phantom. The infusion hose was connected to a medical infusion pump (IV pump, Hospira plum XL series) and the drain hose with connected to a container. It is noteworthy that the height of the drain hose had to be kept at the same height as the infusion port, which was required to maintain the water circulation balance inside the phantom. (C) The assembled water phantom was set between two collimators of the gamma camera during the data acquisition process. (D) Ten consecutive diagrams were acquired directly from the gamma camera scanning. The plot was created from one of the five models derived in this work. The first photo was background scanning before the initial time begun (on the left-side of the first row), the others were taken from 12 minutes of scanning for every 15 minutes within consecutive 2 hour periods.

### Biokinetic model

**Fig 2:**
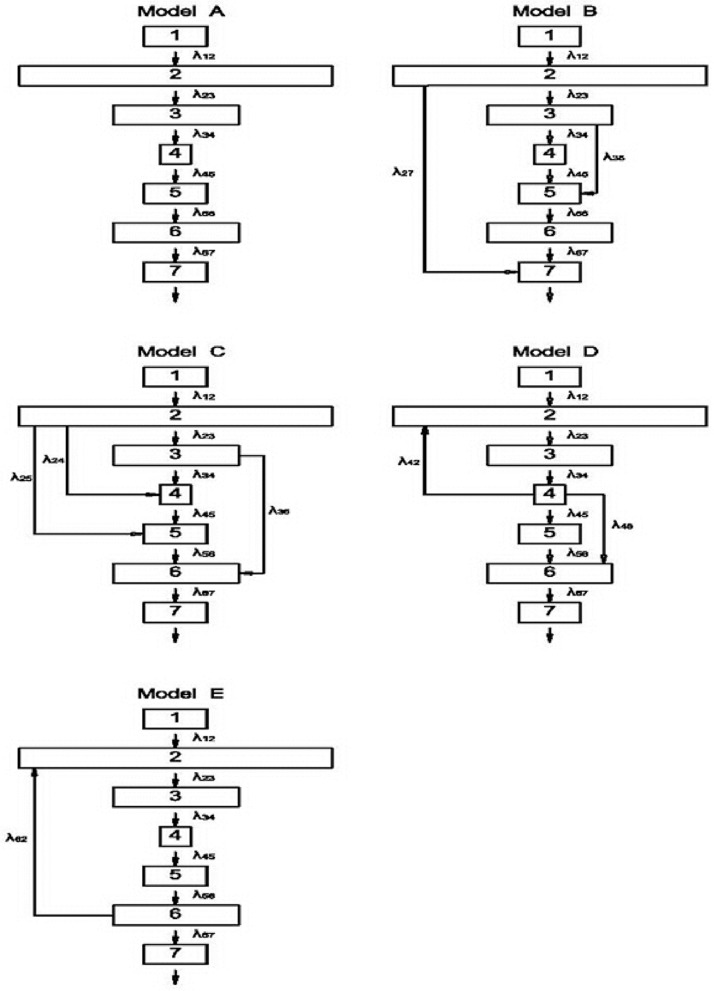
Five biokinetic models were defined using the configuration of seven compartments shown as Regions of Interest (ROIs). The phantom models the different metabolic systems in the human body and each compartment represents a dominant organ (e.g., thyroid, liver, or kidney) or a summarized metabolic system (e.g., the whole body or body fluids).

Five biokinetic models were defined using the configuration of seven compartments shown as Regions of Interest (ROIs) in Fig 2(A)–2(E). The phantom models the different metabolic systems in the human body and each compartment represents a dominant organ (e.g., thyroid, liver, or kidney) or a summarized metabolic system (e.g., the whole body or body fluids) [10]. The phantom can be easily assembled from model A to model E by changing the silicon-based host to a different port connection, as shown in Fig 1B. Eqs 11 through 35 are five groups of simultaneous differential equations that describe the time-dependent correlation among the compartments’ radio-activate solutions.

Model A

$$\frac{dN_{1}}{dt}=-(\lambda_{R}+\lambda_{12})N_{1}$$ (1)

$$\frac{dN_{2}}{dt}=\lambda_{12}N_{1}-(\lambda_{R}+\lambda_{23})N_{2}$$ (2)

$$\frac{dN_{3}}{dt}=\lambda_{23}N_{2}-(\lambda_{R}+\lambda_{34})N_{3}$$ (3)

$$\frac{dN_{4}}{dt}=\lambda_{34}N_{3}-(\lambda_{R}+\lambda_{45})N_{4}$$ (4)

$$\frac{dN_{5}}{dt}=\lambda_{45}N_{4}-(\lambda_{R}+\lambda_{56})N_{5}$$ (5)

$$\frac{dN_{6}}{dt}=\lambda_{56}N_{5}-(\lambda_{R}+\lambda_{67})N_{6}$$ (6)

$$\frac{dN_{7}}{dt}=\lambda_{67}N_{6}-(\lambda_{R}+\lambda_{78})N_{7}$$ (7)

Model B

$$\frac{dN_{1}}{dt}=-(\lambda_{R}+\lambda_{12})N_{1}$$ (8)

$$\frac{dN_{2}}{dt}=\lambda_{12}N_{1}-(\lambda_{R}+\lambda_{23}+\lambda_{27})N_{2}$$ (9)

$$\frac{dN_{3}}{dt}=\lambda_{23}N_{2}-(\lambda_{R}+\lambda_{34}+\lambda_{35})N_{3}$$ (10)

$$\frac{dN_{4}}{dt}=\lambda_{34}N_{3}-(\lambda_{R}+\lambda_{45})N_{4}$$ (11)

$$\frac{dN_{5}}{dt}=\lambda_{45}N_{4}+\lambda_{35}N_{3}-(\lambda_{R}+\lambda_{56})N_{5}$$ (12)

$$\frac{dN_{6}}{dt}=\lambda_{56}N_{5}-(\lambda_{R}+\lambda_{67})N_{6\;}$$ (13)

$$\frac{dN_{7}}{dt}=\lambda_{67}N_{6}+\lambda_{27}N_{2}-(\lambda_{R}+\lambda_{78})N_{7}$$ (14)

Model C

$$\frac{dN_{1}}{dt}=-(\lambda_{R}+\lambda_{12})N_{1}$$ (15)

$$\frac{dN_{2}}{dt}=\lambda_{12}N_{1}-(\lambda_{R}+\lambda_{23}+\lambda_{24}+\lambda_{25})N_{2}$$ (16)

$$\frac{dN_{3}}{dt}=\lambda_{23}N_{2}-(\lambda_{R}+\lambda_{34}+\lambda_{36})N_{3}$$ (17)

$$\frac{dN_{4}}{dt}=\lambda_{34}N_{3}+\lambda_{24}N_{2}-(\lambda_{R}+\lambda_{45})N_{4}$$ (18)

$$\frac{dN_{5}}{dt}=\lambda_{45}N_{4}+\lambda_{25}N_{2}-(\lambda_{R}+\lambda_{56})N_{5}$$ (19)

$$\frac{dN_{6}}{dt}=\lambda_{56}N_{5}+\lambda_{36}N_{3}-(\lambda_{R}+\lambda_{67})N_{6}$$ (20)

$$\frac{dN_{7}}{dt}=\lambda_{67}N_{6}-(\lambda_{R}+\lambda_{78})N_{7}$$ (21)

Model D

$$\frac{dN_{1}}{dt}=-(\lambda_{R}+\lambda_{12})N_{1}$$ (22)

$$\frac{dN_{2}}{dt}=\lambda_{12}N_{1}+\lambda_{42}N_{4}-(\lambda_{R}+\lambda_{23}+\lambda_{26})N_{2}$$ (23)

$$\frac{dN_{3}}{dt}=\lambda_{23}N_{2}-(\lambda_{R}+\lambda_{34})N_{3}$$ (24)

$$\frac{dN_{4}}{dt}=\lambda_{34}N_{3}-(\lambda_{R}+\lambda_{45}+\lambda_{42})N_{4}$$ (25)

$$\frac{dN_{5}}{dt}=\lambda_{45}N_{4}-(\lambda_{R}+\lambda_{56})N_{5}$$ (26)

$$\frac{dN_{6}}{dt}=\lambda_{56}N_{5}+\lambda_{26}N_{2}-(\lambda_{R}+\lambda_{67})N_{6}$$ (27)

$$\frac{dN_{7}}{dt}=\lambda_{67}N_{6}-(\lambda_{R}+\lambda_{78})N_{7}$$ (28)

Model F

$$\frac{dN_{1}}{dt}=-(\lambda_{R}+\lambda_{12})N_{1}$$ (29)

$$\frac{dN_{2}}{dt}=\lambda_{12}N_{1}+\lambda_{62}N_{6}-(\lambda_{R}+\lambda_{23})N_{2}$$ (30)

$$\frac{dN_{3}}{dt}=\lambda_{23}N_{2}-(\lambda_{R}+\lambda_{34})N_{3}$$ (31)

$$\frac{dN_{4}}{dt}=\lambda_{34}N_{3}-(\lambda_{R}+\lambda_{45})N_{4}$$ (32)

$$\frac{dN_{5}}{dt}=\lambda_{45}N_{4}-(\lambda_{R}+\lambda_{56})N_{5}$$ (33)

$$\frac{dN_{6}}{dt}=\lambda_{56}N_{5}-(\lambda_{R}+\lambda_{67}+\lambda_{62})N_{6}$$ (34)

$$\frac{dN_{7}}{dt}=\lambda_{67}N_{6}-(\lambda_{R}+\lambda_{78})N_{7}$$ (35)

The terms N_i_ and λ_ij_ represent, respectively, the time-dependent quantity of the specific radionuclide and the biological decay constant (λ_ij_ = i_ij_·ln2/T_i(1/2)_(bio), where i_ij_ is the branching ratio of the infused water from the i^th^ to the j^th^ compartment, T_i(1/2)_(bio) is the biological half-life of the i^th^ compartment, and λ_R_ is the radiological decay constant of the specific radionuclide, which, in this work, is Tc-99m. In model A, only one path was preset from ROI (1) to ROI (7); therefore, in the theoretical calculation, i_ij_ is always 1.0. Meanwhile, in models B-E, the infused water was split into two or three paths either in the beginning (model B, C) or in the middle stage (model D, E), making the sum of i_ij_ equal 1.0 ($\Sigma_{j=1}^{n}i_{ij}=1.0$) of the model computation process. For example, in model B, compartment 1 had only one path to compartment 2 (i_12_ = 1.0), while compartment 2 was split into two paths (i_23_+i_27_ = 1.0) into compartments 3 and 7. A self-developed program in MATLAB was used to simulate and plot the results validating the optimal solution for Eqs 1–35, as well as to plot the time-dependent simultaneous differential equations to verify the specific biokinetic models as assigned in this work. The algorithm of the program was developed by the inverse matrix analysis to acquire a numerical solution as implied in Eqs (36) and (37). The parameters as listed in Table 1 were reorganized into a coefficient matrix M_ij_ [7×7] for solving Eqs 1–35 in each model.

[dNidt]=[Mij]∙[Nj]; i=j=7, thus,

 [Mij]-1∙ [dNidt] = [Mij]-1∙ [Mij]∙[Nj]=I7 ∙ [Nj] (36)

[7×1]=[7×7]∙[7×1]=[7×1] (37)

### Gamma camera survey

This study used 129.1±24.2 MBq of Tc-99m labeled methylene diphosphonate (MDP) in five continuous scans [11]. The activated solution was mixed homogeneously with 1610 c.c. of 0.9% saline solution that was diluted using pure deionized water and infused into the dynamic phantom before measurement. The 0.9% saline solution provided similar density to Tc-99m-MDP and was essential to ensure balance within the mixed solution. A medical infusion pump (IV pump, Hospira plum XL series) was connected to the infusion port (see Fig 1B, on the left side of the phantom) with a silicon-based 2.0-m hose, and the drain port was attached to a 1.5-m hose. The crucial factor in this arrangement was that the maximum height of the drain hose needed to be the same height as the infusion port; this was essential to maintaining water balance inside the phantom (see Fig 1B, on the right side of the phantom).

An initial time measurement was recorded when the IV pump infused the fresh water into the phantom. The theoretical biological half-life of every assigned box (*i*.*e*., defined as compartment through Eqs 1–35) was defined as half of the volume divided by the feed-water infusion rate. For example, the biological half-life of box 1 (ROI (1)) was 10.7 min when the feed-water infusion rate was set at 6.7 c.c./min (144 [c.c.] / 2 / 6.7 [c.c.·min.^-1^] = 10.7 [min]). Further, λ_12_ for model A (see Eqs 1 and 2) was determined to be 0.0648 [min^-1^] (ln2/10.7 = 0.0648) when i_12_ was 1.0. λ_R_ is 0.001925 [min^-1^] (ln2/360 = 0.001925) because the radiological half-life of Tc-99m is 360 min. However, the adjustment of practical biological half-life for every ROI was allowed to satisfy the real measured data in the gamma camera image acquiring system.

The gamma camera (Infinia Hawkeye 4) used in this experiment was located at the Department of Nuclear Medicine in Buddhist Tzu Chi General Hospital, Taichung. During scanning, the camera’s two NaI (54×40×0.95 cm^3^) plate detectors were positioned 10 cm in front of and 12 cm behind the phantom. To record data, each plate was connected to a group of 53×3 in- and 6×1.5 in-diameter photo multiplier tubes (PMT). The two detectors captured approximately 70% of the emitted gamma rays that were behind the low energy high resolution (LEHR) collimator [2, 12]. Fig 1C shows the arrangement of the water phantom between the gamma camera’s two NaI plates. As it has been noted, to assure water balance the container holding the drained water was kept at the same height as the top of the phantom.

**Fig 3:**
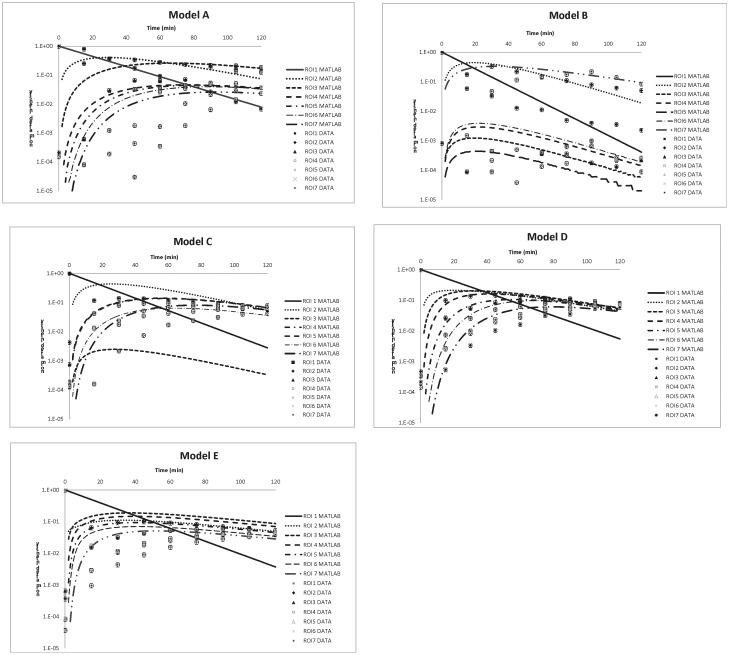
The results of the five models obtained from MATLAB simulation in the form of seven continuous lines, as well as the normalized data set in seven groups of divided dots.

Data collection and measurement were initiated when the IV pump began to feed in the water. The scan protocol consisted of a 12 minute-collection, every 15 minutes, for two consecutive hours and consisted of practical measurements for vertical position, energy peak of 140 keV (window: 25%), LEHR collimator, 128×128 matrix, and static scan. Data sets were obtained and analyzed for each case as shown in Fig 1D. Well-trained radiologists marked and recorded the ROIs directly from the clinical monitor next to the gamma camera facility. For obtaining the practical dataset, collected data of the time-dependent counts/pixel/sec for each specific ROI, that were recorded directly from the gamma camera data acquiring system (cf. Fig 1D), were normalized to the primary time-zero one of ROI(1) (cf. Fig 2 model A-E). Individually, the normalization process in every model was accomplished by dividing every recorded time-dependent counts/pixel/sec in ROIs(1–7) by the maximal one, *i*.*e*., ROI(1) at time-zero. This is essential to integrate the empirical data (separated dots in Fig 3) with theoretical prediction from MATLAB program (continuous lines in Fig 3), whereas the theoretical evaluation was done by optimizing the preset parameters as coefficient matrix M_ij_ [7×7] (cf. Eqs 36 and 37, Table 1). Also, the output from MATLAB program provided a converged approach with a close correlation for all time-dependent ROIs, so that no single ROI could be adjusted separately. Restated, the coefficient matrix M_ij_ [7×7] had to be considered thoroughly to fulfill the theoretical definitions in each model. Thus, an optimal coefficient matrix can significantly suppress the difference between theoretical predictions and empirical data.

**Table 1:** The derived biological half-lives and variables for the simultaneous differential equations used in this work. The calculation results represent theoretical estimations of the time-dependent quantity of Tc-99m in various boxes for all of the models, as determined by the MATLAB calculation. Tc-99m’s physical half-life is indicated as T_1/2_(R), which is 6.02 h, i.e., 360 min, approximately.

	Single Path			With feedback		Theoretical Derivation [min]
	A	B	C	D	E	
T_1/2_(R)	360	360	360	360	360	360
T_1/2_(ROI 1)	15.0	13.0	12.8	16.7	11.7	144/2/6.7 = 10.7
T_1/2_(ROI 2)	30.0	18.0	23.5	32.8	31.5	612/2/6.7 = 45.5
T_1/2_(ROI 3)	13.8	4.8	4.4	15.6	12.8	252/2/6.7 = 18.8
T_1/2_(ROI 4)	5.0	5.0	6.8	4.8	11.8	64/2/6.7 = 4.8
T_1/2_(ROI 5)	9.0	7.5	6.6	8.7	10.7	144/2/6.7 = 10.7
T_1/2_(ROI 6)	14.5	11.5	5.8	8.8	9.8	252/2/6.7 = 18.8
T_1/2_(ROI 7)	3.0	35.7	7.7	5.7	10.7	144/2/6.7 = 10.7
i_12_	1.0	1.0	1.0	1.0	1.0	
i_23_	1.0	0.2	0.1	1.0	0.3	
i_34_	0.5	0.4	0.8	1.0	1.0	
i_45_	1.0	1.0	1.0	0.3	1.0	
i_56_	1.0	1.0	0.05	1.0	1.0	
i_67_	1.0	1.0	1.0	1.0	0.1	
i_27_		0.8				
i_35_		0.6				
i_24_			0.4			
i_25_			0.5			
i_36_			0.2			
i_42_				0.4		
i_46_				0.3		
i_62_					0.9	

## Results

### Biokinetic models

The time-dependent biokinetic model can significantly help to interpret quantity changes in various compartments. Models that have not been widely reported can be practically verified through indigenous phantoms. This study surveyed and analyzed five cases (models A to E) from many possible combinations by the assembled phantoms. Three of these were single path models (models A, B, and C) and two had feedback (models D and E). The feedback loop was created using a hose with a one-way value. Fig 3 shows the results of the five models obtained from MATLAB simulation in the form of seven continuous lines, as well as the normalized data set in seven groups of divided dots. Table 1 shows all of the derived biological half-lives and variables for the simultaneous differential equations used in this work. The calculation results represent theoretical estimations of the time-dependent quantity of Tc-99m in various boxes for all of the models, as determined by the MATLAB calculation. Tc-99m’s physical half-life is indicated as T_1/2_(R), which is 6.02 h, *i*.*e*., 360 min, approximately. Most of the ROIs’ derived biological half-lives were not in agreement with the theoretical ones, which were calculated as half the specific ROI volume divided by the feed-water flow rate (6.7 c.c./ min.). The derived biological half-life of ROI(1) in all models (11.7–15.0 min.) was mostly in agreement with the theoretical one (10.7 min.) since only one output port was connected from ROI(1) to (2) in all of the models. In contrast, the derived biological half-lives of the remaining ROIs in all models fluctuated significantly from the preset half-lives. The different connections among the various boxes in all the models also created large complexity. Individually, in models B and C, box two was connected to two or three output ports, whereas in models D and E, the feedback water was reinfused from box 4 or box 6 to box 2 (see Fig 2). The derived branching ration i_ij_ was not unified even if it was preset as a single input or output in the biokinetic model (e.g., i_34_ of Model A, or i_56_ of Model C, see Fig 2). This might be the radioactive solution deposited beneath the box and barely washed out by the infused water; however, the optimal solution from MATLAB reflected the real solution determined during gamma camera scanning. The derived data of models A-E were implied thoroughly in S1–S5 Tables.

### AT examination

The scanned data from the gamma camera for all models were collected, converted to count/pixel/sec then normalized. An agreement value (AT) was used to determine the similarity between the optimal predictions obtained by MATLAB and the empirical data of every box for all models and a weighted AT (AT_W*i*_) was summarized to judge the performance of the derived results for each specific model. The AT was revised from the root mean square (RMS) in evaluating a significant amount of acquired data [13, 14]. AT and weighted AT are both defined below.

$${AT}_{i}=\sqrt{\frac{\sum_{i\;=\;1}^{n}{\left(\frac{Y_{i}(Matlab)-Y_{i}(nor.)}{Y_{i}(nor.)}\right)}^{2}}{N}\;}\;\times 100\%$$ (38)

ATWi=∑17 Voli ∙ATi∑17Voli (39)

where Y_i_ (nor.) and Y_i_(Matlab) were the normalized intensity from each ROI determined from the n_th_ set of empirically obtained data and were each computed using MATLAB. The value of N was set at 9 so that every fifteen minutes represented a point from the interpolation of the practical data set. An AT of zero suggested a perfect agreement between the theoretical and empirical results. An AT under 20 can be regarded as indicating the excellent consistency between the optimal computational and empirical data. This was derived on the basis that empirical data fluctuated averagely among ±20% to the computational estimations. An AT between 20 and 50 supported a reasonable confidence in the consistency since the AT was obtained point by point by comparing the practical data to the theoretical calculation results for each ROI [5, 15, 16]. A large ATs always corresponds to low layers of ROIs as is shown in Fig 2, i.e., ROIs(4–7), implied that cumulative uncertainties were piled up gradually from high to low ones, and, also, a complicate connection of the linked path in model C or D was also related to large ATs. Meanwhile, AT_wi_ was defined as a weighted average of ATs in one specific model. A sizeable weighted factor was assigned to a large volume box in calculating the AT_Wi_ since it dominated the performance of time-dependent correlations among boxes. Table 2 displays the ATs and AT_Wi_ for the five models. The AT indicates the curve fitting agreement between the theoretical estimation and practical scanned for every specific ROI. Either 10 or 11 of all thirty-five (7×5 = 35) ATs were between 0–20 or 21–50. Moreover, the AT_Wi_ also fluctuated from 23 to 53, indicating that the quantitative study is acceptable.

**Table 2:** The ATs and AT_Wi_ for the five models. The AT indicates the curve-fitting agreement between the theoretical estimation and practical scanned for every specific ROI.

	Single path			With feedback	
	A	B	C	D	E
ROI (1)	20	26	5	30	2
ROI (2)	32	41	45	31	19
ROI (3)	61	2	65	47	48
ROI (4)	81	3	20	54	45
ROI (5)	95	1	56	57	61
ROI (6)	72	5	56	50	58
ROI (7)	71	44	13	47	63
**AT**_**Wi**_	**53**	**23**	**44**	**42**	**37**

## Discussion

### Model interpretation

Model A can be interpreted as a series of continuous chain decays of the Tc-99m radio-activated solution since there is no other branch that can bypass the feed-water. The infuse water flows directly from ROI(1) to ROI(7). ROI(2) has the largest volume, resulting in a correlation between ROI(1) and ROI(2) defined similarly to “no equilibrium” in the chain decay series. The concentration in ROI(1) is degraded rapidly to ROI(2), whereas ROI(2) and ROI(3) can be considered to be at “transient equilibrium.” The maximum concentration of Tc-99m in ROI(3) can be calculated as approximately 28.6 min. (T_max_ = (Lnλ_2_ –Lnλ_1_) / (λ_2_-λ_1_) = 28.6 min.) [17]. The recorded counts reach the highest point when real scanning begins after 30 min, and in the MATLAB simulation after 29 min (see Fig 3, model A). The time to reach maximum concentration (T_max_) for the following ROIs can be readily derived from the MATLAB calculations as 66, 74, 82, 89, and 97 min for ROIs (3)-(7), respectively. The highest AT among the ROIs is 95, which was obtained from ROI(5) and resulted in the highest AT_Wi_, 53 in all models (see Table 2). It is noteworthy that the largest ROI(2) tactically dominates the chain correlation among all ROIs; therefore, any interpretation of an individual ROI’s performance might mislead the integrated performance. A weighted AT, AT_Wi_, is, therefore, proposed to solidify the verification.

Model B, ROI(2) has two output ports to ROI(3) and (7), and ROI(3) has two output ports to ROI(4) and (5) (see Fig 2, model B). The sum of i_23_ and i_27_ (or i_34_ and i_35_) is equal to unity (0.2+0.8 or 0.4+0.6, see Table 1, model B), which indicates that there is no significant deposition of activated solution observed either in gamma scanning or the MATLAB optimal regression fit. When compared to the theoretical settings, the low biological half-life derived from ROI(2), (3), (5), or (6) and the high biological half-life derived from ROI(7) do not necessarily suggest an inaccurate approach in this work, because one additional hose links ROI(2) to (7), and 80% (i.e. i_27_ = 0.8) of the infused water directly bypassed from ROI(2) to (7). Furthermore, ROI(3) has two outputs to either (4) or (5), which changes the original setting that was primarily preset according to model A. The biological half-life in every box of all of the models is not merely derived for satisfying a single group of individual data dots: it holds for all seven groups of data dots. This is accomplished to ensure a minimum weighted AT (AT_Wi_); therefore, a small ROI volume may be concluded as an unrealistic option for compromising the optimal solution in the whole system. Additionally, in the MATLAB program, the optimal solution is defined via the time-dependent simultaneous differential Eqs 1–35 approaching the lowest AT_Wi_, Here ROI(2) has the most extensive volume, while ROI(3), (6) share the second place. Therefore, the optimal solution obtained from the calculation is biased toward ROI(2), (3) or (6); and the minor ones (ROI(1), (4), (5) or (7)) are ignored because, theoretically, they have a small volume.

Of the seven boxes in this work, Model C has the most complicated hose-connections. The three and two output ports of ROI(2) and (3), respectively, cause the derived biological half-lives to be much shorter than the theoretical settings (practical 23.5 and 4.4 min. compared to theoretical 45.5 and 18.8 min.). In contrast, the more extended half-lives reported in ROI(4) (6.8 to 4.8) reflect a reasonable finding that an additional feed-water is infused directly toward ROI(4) to hold the decay of Tc-99m inside the water box. The cross-interactions between individual boxes are too complicated to be interpreted individually via this model. However, the compromised solution provided by the MATLAB program herein can support a mathematical suggestion with a converged and acceptable consequent.

Model D and model E both have a feedback hose connection; therefore, the feed-water is only able to be reinfused from ROI(4) to ROI(2) or from ROI(6) to (2). In model D, the smallest volume of ROI(4) is equally distributed to the feed-water in three branches (i_42_, i_45_, and i_46_), whereas in model E, nearly 90% of the feed-water is reinfused from ROI(6) to (2) (i.e., i_62_ = 0.9) and the branch ratios are verified from the MATLAB optimal suggestion. However, the practical data recording does not support this setting, although the theoretical preset is equally distributed in the initial stage of calculation. It should be noted that the AT of ROI(4) in model D is acceptable but high, 54 implies the time-dependent activity change. Nonetheless, the ROI(4) is the most crucial because it links one input, one feedback, and two output hoses in the system default. The specific AT of ROI(4) can be efficiently reduced if the other parameters are revised in the MATLAB calculation. However, the smallest volume of ROI(4) restrains its contribution in defining the AT_Wi_. The AT_4_ in model D is, therefore, compromised in the theoretical evaluation.

In contrast to model D, model E has a simple water loop. Only one feedback path is preset from ROI(6) back to (2), and the derived AT_Wi_ is 37, which is better than the derived AT_Wi_ in model D, 42. In practical application, both the iodine thyroid model and the gastrointestinal tract (GI Tract) model have a feedback connection [2, 16]. The feedback path is frequently defined in biokinetic models to suggest a compartment, such as the liver or kidney, with a unique metabolic mechanism in the human body. In contrast, few works have been reported in this field.

### Model application

**Fig 4:**
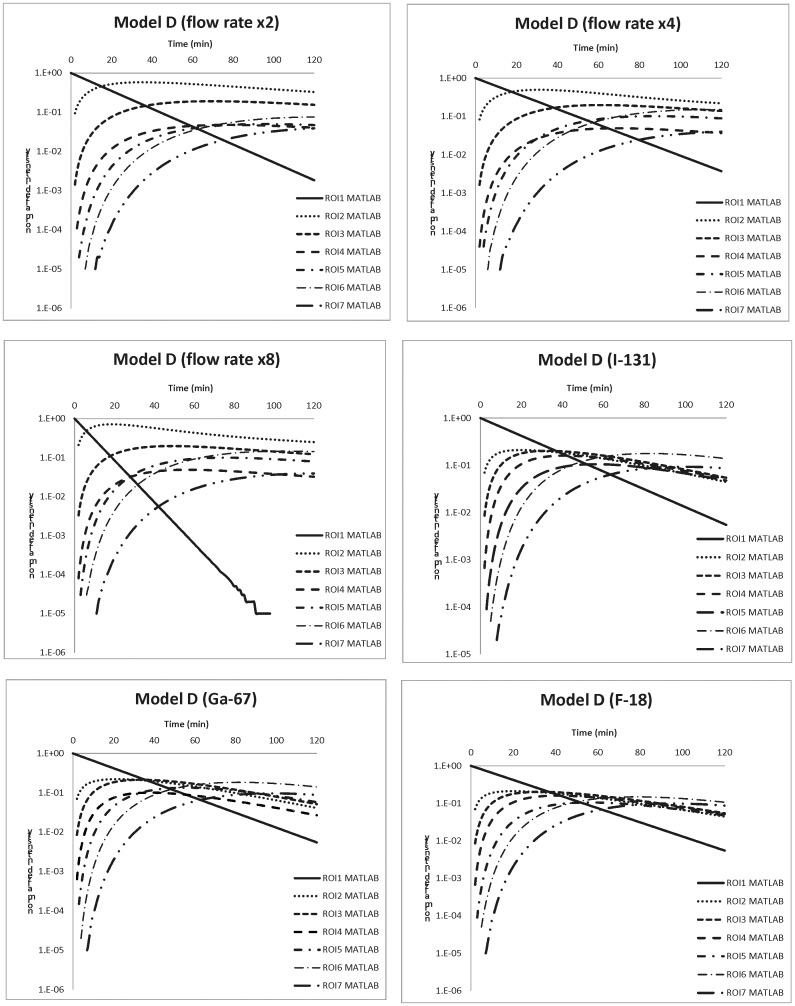
Six MATLAB calculation results using different settings. In the first three plots, the labeled radionuclide is still Tc-99m but the feed water infusion rate is two, four or eight times faster than the original setting, 6.7 c.c./min, and in the last three plots Tc-99m is replaced by I-131 (T_1/2_(R) = 8.03 d), Ga-67 (T_1/2_(R) = 78 h) and F-18 (T_1/2_(R) = 110 m), respectively.

A thorough evaluation of biokinetic models confirms the correlation between dose administration and the organ equivalent dose for patients who either undergo a nuclear examination or get radiopharmaceutical medicines. The preset radionuclide Tc-99m can be replaced with any other radionuclide in the MATLAB program. This is convenient for researchers or medical professionals who wish to explore the applications of this technique to other medicinal purposes. Fig 4 depicts six MATLAB calculation results using different settings. In the first three plots, the labeled radionuclide is still Tc-99m but the feed-water infusion rate is two, four or eight times faster than the original setting, 6.7 c.c./min, and in the last three plots Tc-99m is replaced by I-131 (T_1/2_(R) = 8.03 day), Ga-67 (T_1/2_(R) = 78 h) and F-18 (T_1/2_(R) = 110 min), respectively. As Fig 4 illustrates, the last three plots perform similarly about the time-dependent intensity of ROIs, because the preset biological half-life of every ROI dominates the curve characteristics. A radiological half-life of either 8.03 day, 78 h or 110 min has a negligible contribution to the time-dependent intensity change; in contrast, the biological half-life of the seven ROIs that varies from 6.7 min to 3.4, 1.7, or 0.84 min in the original setting reflects a significant change. When the feed-water infusion rate increases from original to multiple folds, the predicted results become remarkably different, because the radioactive degradation in ROI (1) also falls at numerous folds of the preset rate. ROIs (2) to (7) change significantly according to the cross interaction among ROIs.

## Conclusions

This study presents a quantitative evaluation of five biokinetic models with their verification by joint theoretical simulation and practical measurement. The results derived from the five biokinetic models provided both a theoretical basis through running the program in MATLAB and also an analytical approach using gamma camera scanning with derived weighted AT that ranged from 23 to 57. The dimensionless AT_i_ or AT_Wi_ provided a quantitative index for validating the integrated comparative analysis of the model theoretical simulation and practical evaluation. The feasible multiple-box water phantom is constructed according to the user’s definition to imply realistic, unique demands. The derived biokinetic model was proved to accurately represent the metabolic mechanism in the human body and to be instrumental in solidifying the internal circulatory system into reality with numerical simulation verification.

## Supporting information

S1 Table The derived data of model A. (XLSX)

S2 Table The derived data of model B. (XLSX)

S3 Table The derived data of model C. (XLSX)

S4 Table The derived data of model D. (XLSX)

S5 Table The derived data of model E. (XLSX)
